# Prolonged partial upper airway obstruction during sleep – an underdiagnosed phenotype of sleep-disordered breathing

**DOI:** 10.3402/ecrj.v3.31806

**Published:** 2016-09-06

**Authors:** Ulla Anttalainen, Mirja Tenhunen, Ville Rimpilä, Olli Polo, Esa Rauhala, Sari-Leena Himanen, Tarja Saaresranta

**Affiliations:** 1 Division of Medicine, Department of Pulmonary Diseases, Turku University Hospital, Turku, Finland; 2 Department of Pulmonary Diseases and Clinical Allergology, University of Turku, Turku, Finland; 3 Sleep Research Centre, Department of Physiology, University of Turku, Turku, Finland; 4 Department of Clinical Neurophysiology, Medical Imaging Centre and Hospital Pharmacy, Pirkanmaa Hospital District, Tampere University Hospital, Tampere, Finland; 5 Department of Medical Physics, Medical Imaging Centre and Hospital Pharmacy, Pirkanmaa Hospital District, Tampere University Hospital, Tampere, Finland; 6 School of Medicine, University of Tampere, Tampere, Finland; 7 Unesta Research Center, Tampere, Finland; 8 Department of Pulmonary Diseases, Tampere University Hospital, Tampere, Finland; 9 Department of Clinical Neurophysiology, Satakunta Hospital District, Pori, Finland

**Keywords:** prolonged partial upper airway obstruction, flow limitation, increased respiratory resistance, non-apneic snoring, simple snoring, sleep, sleep apnea, UARS

## Abstract

Obstructive sleep apnea syndrome (OSAS) is a well-recognized disorder conventionally diagnosed with an elevated apnea–hypopnea index. Prolonged partial upper airway obstruction is a common phenotype of sleep-disordered breathing (SDB), which however is still largely underreported. The major reasons for this are that cyclic breathing pattern coupled with arousals and arterial oxyhemoglobin saturation are easy to detect and considered more important than prolonged episodes of increased respiratory effort with increased levels of carbon dioxide in the absence of cycling breathing pattern and repetitive arousals. There is also a growing body of evidence that prolonged partial obstruction is a clinically significant form of SDB, which is associated with symptoms and co-morbidities which may partially differ from those associated with OSAS. Partial upper airway obstruction is most prevalent in women, and it is treatable with the nasal continuous positive pressure device with good adherence to therapy. This review describes the characteristics of prolonged partial upper airway obstruction during sleep in terms of diagnostics, pathophysiology, clinical presentation, and comorbidity to improve recognition of this phenotype and its timely and appropriate treatment.

Obstructive sleep apnea syndrome (OSAS) is a common clinical disorder, which is associated with a reduced quality of life, as well as significant comorbidity and mortality (1–5). In spite of the growing knowledge that upper airway obstruction during sleep may be either complete or partial and prolonged, nearly all studies use apnea–hypopnea index (AHI) to classify the severity of sleep-disordered breathing (SDB). AHI measures the frequency of periodic obstructive or central events per hour of sleep. Identification of apneic events as obstructive, central or mixed relies on strain gauges, oronasal thermistors or monitoring inspiratory flow via nasal pressure transducer (6). The AHI has been criticized for 30 years for oversimplification of SDB (7). Prolonged upper airway obstruction or increased respiratory resistance (IRR) without significant hypopnea or desaturation is not considered. This may result in ignorance of a significant time of the recordings when the subject increases his or her respiratory efforts to fight against prolonged partial upper airway obstruction. It is known that prolonged partial upper airway obstruction may result in daytime symptoms and increase the risk of developing high blood pressure by 42% even if AHI remains normal (8–11). Partial upper airway obstruction is the most common form of SDB in females (10, 12, 13). It is important to recognize partial upper airway obstruction and agree about the standards of its assessment in order to build sufficient body of evidence for the pathophysiological and clinical significance of this phenotype of SDB.

Partial upper airway obstruction is underrepresented in the literature for many reasons. First, there is no consensus of its detection, quantification or clinical impact. Second, episodes of partial upper airway obstruction are usually prolonged, typically for several minutes, and the severity of which cannot be defined on an event basis [as is done with AHI or respiratory disturbance index (RDI)]. Since patients with prolonged partial upper airway obstruction may have low AHI, it is commonly interpreted as mild sleep apnea, not warranting treatment. Third, partial upper airway obstruction is associated with increased partial pressure of carbon dioxide (CO_2_) (14, 15), which is rarely measured in the context of a sleep study. Finally, part of the ignorance can be attributed to the fact that arousals during partial obstruction are not as frequent as they are during repetitive episodes of sleep apnea (16). Despite the lack of solid correlation between the arousal frequency and excessive sleepiness (17), the cardinal symptom of SDB, other mechanisms than arousals or sleep fragmentation is rarely considered behind sleepiness. There is an urgent need to recognize partial obstruction as a treatable form of SDB in order to understand why certain patients with low AHI may still suffer from symptoms and benefit from appropriate intervention. This review is aiming to increase awareness and recognition of prolonged, sustained partial upper airway obstruction during sleep as a phenotype of SDB.

## Pathophysiology of prolonged partial upper airway obstruction

The upper airway is often modeled as a collapsible tube, the patency of which is critically dependent on the upper airway dilator muscle tone (18). Transition from wakefulness to sleep is associated with decreasing sympathetic tone (19) and decreasing upper airway muscle tone (20) and increased upper airway resistance (21), which are the reasons why SDB is sleep state dependent. Normally, simultaneously decreasing sympathetic activity allows for a physiologic increase in CO_2_, which has a stabilizing effect on the upper airway (22). In other words, in healthy individuals, during transition from wakefulness to sleep, the upper airway dilating forces shift from the muscle tone of wakefulness to muscle support by physiologically increased levels of CO_2_ occurring during stable sleep.

In patients with obstructive sleep apnea or hypopnea, this upper airway stabilizing process of increasing CO_2_ level is repetitively interrupted. Structurally too-narrow airways may collapse completely, and an arousal is required to restore the sympathetic tone and upper airway muscle tone. This results in hyperventilation, decreased levels of CO_2_, instability of the upper airway and instability of the breathing pattern (cyclic breathing). The repetitive pattern allows for counting the events per hour (apnea index, AHI or RDI).

During prolonged partial upper airway obstruction, the inspiratory duty cycle (Ti/Ttot) is prolonged (23–25) and breathing frequency increased (23). Arousals are less frequent during sleep induction, which allows for CO_2_ to increase until stable sleep is established. If the upper airway still remains partially obstructed, stability between the collapsing and dilating forces may lead to CO_2_ increase at much higher levels than would be optimal for the system during sleep [the plateau concept (26)]. As a result, the intensity of respiratory effort and the intrathoracic pressure swings may increase in a crescendo pattern until stabilizing for long periods above the optimal level of CO_2_ during sleep or terminating in arousal.

Collapsible properties as well as neuromuscular responses of the upper airway have been studied by applying negative pressure to the upper airway and determining the critical closing pressure (Pcrit) (18, 27, 28). It has been suggested that the upper airway collapsibility is a continuum from normal breathing to snoring, hypopnea and finally apnea and that it associates with increasing Pcrit (28). A recent study showed that Pcrit and genioglossus muscle activity are sleep stage dependent and that muscle activity is greatest during slow-wave sleep (SWS) (20). OSA patients and non-apneic snorers have also an increased threshold for detecting vibration in the upper airway which could predispose upper airway obstruction (29). This impairment is partially reversible with continuous positive airway pressure (CPAP), suggesting that upper airway sensory function deteriorates as the disease progress.

**Fig. 1:**
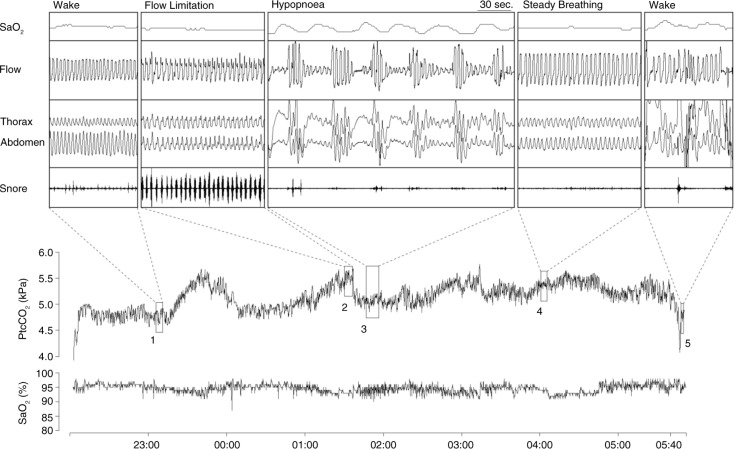
Example of overnight PtcCO_2_ and SaO_2_ profile with expanded view of normal steady breathing, prolonged flow limitation and hypopnea sequence. Note the association between breathing type and PtcCO_2_ (15). (Reproduced with permission from Respir Physiol Neurobiol). PtcCO_2_=transcutaneous carbon dioxide; SaO_2_=arterial oxyhemoglobin saturation.

Prolonged partial upper airway obstruction represents a situation where balance is established between collapsing and dilating forces. This compensation however is not complete. It is sufficient enough to prevent the development of hypoxia to a level which would strongly stimulate the breathing, but few studies have shown that these episodes associate with increased CO_2_ levels (14, 15, 25, 30) suggesting insufficient compensation regarding the CO_2_ (Fig. 1). In a recent study inspiratory flow limitation episodes were associated with 0.2 kPa PtcCO_2_ increase from normal breathing levels (15). Study by Lo et al. (22) showed that when inspiratory CO_2_ is increased (5 or 10 mmHg) above the eupneic level, increase in genioglossus EMG activity is observed. Unloading the upper airway with CPAP did not alter the slope of genioglossus activity although the overall activity was lower. This implies that both chemoreceptor as well as negative pressure reflex modulates the upper airway patency.

## Diagnostics of prolonged partial upper airway obstruction during sleep

**Fig. 2:**
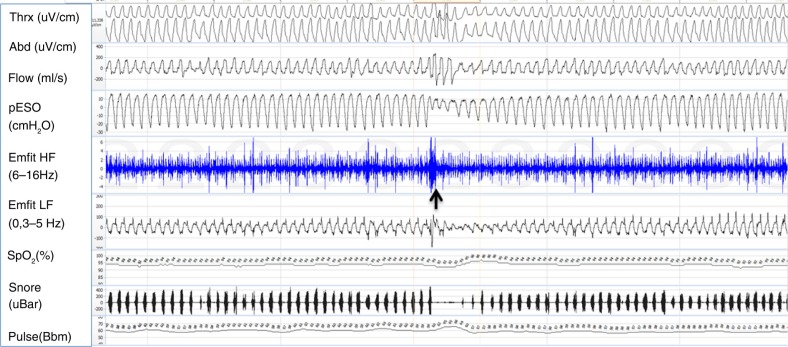
Example of a 5-min polysomnography period. At the beginning of the sheet, respiratory movements are stable; flow channel shows slight flow limitation and mouth breathing. Negative esophageal pressure is increased up to −30 cm H_2_O. Emfit high-frequency channel shows multiple spikes. At the middle of sheet (marked with a black arrow) is a short arousal with opening of upper airway, normalizing esophageal pressure values and cease of spiking. Gradually breathing effort starts to increase again. Channels from top: thoracic and abdominal belts, flow by nasal pressure transducer, esophageal pressure, Emfit high-frequency channel, Emfit low-frequency channel, arterial oxyhemoglobin saturation, snoring, and pulse.

Upper airway dysfunction during sleep has been described with variable terminology in the literature (Table 1). Standardized diagnostic criteria for prolonged partial upper airway obstruction do not exist. In clinical practice, periods longer than hypopnea (minimum 1–3 min) are often used as indicative of sustained upper airway resistance (10, 31). During partial obstruction breathing, effort prevails against partially closed upper airway resulting in diminished airflow, which can be measured reliably by pneumotachograph. Even if it is a valid method to quantify partial obstruction, nasal prongs or cannula revealing inspiratory flow limitation are often used as a surrogate of the pneumotachographic signal in clinical sleep studies. Measuring increasing effort provides another reliable means to evaluate the increased resistance. The golden standard to assess inspiratory effort is measuring esophageal pressure (pESO) (6). In pESO signal the prolonged partial obstruction induces episodes of sustained increased negativity without periodic arousals (Fig. 2). The pattern is clearly distinguishable from the periodic pESO swings, which terminate in arousals and are related to apneas, hypopneas and UARS (Upper Airway Resistance Syndrome) events. However, the pressure catheter is often considered uncomfortable and in clinical work indirect, non-invasive methods are preferred.

**Table 1:** Definitions and special characteristics of the various terminologies that have been used to describe upper airway dysfunction during sleep

			Prolonged partial obstruction	
			
	OSA	UARS	IRR	Flow limitation
Key sensors	Thermistors, nasal prongs connected to pressure transducer and thoracic/abdominal belts	Esophageal pressure catheter and polysomnography (RERAs with EEG)	Sleep mattress and esophageal pressure	Nasal prongs connected to pressure transducer
Patient's respiratory efforts	Periodic breathing (periodic increases in respiratory efforts)	Increased for few breaths	Increased for prolonged periods (1 to over 60 min)	Normal or increased
Arousals	Repetitive	Repetitive, terminate each event	Terminates episodes, association to respiration unambiguous	May terminate episodes
SaO_2_	Repetitive dips	No change	No change or slow desaturation	No change or slow desaturation
CO_2_	Oscillation	No change	Gradual retention	No change or gradual retention

OSA=obstructive sleep apnea, UARS=upper airway resistance syndrome, IRR=increased respiratory resistance, RERA=respiratory effort-related arousal, EEG=electroencephalogram, SaO_2_=arterial oxygen saturation, CO_2_=carbon dioxide.

In routine clinical polysomnography (PSG) the nasal pressure transducer signal provides an easy method to evaluate the amount of prolonged partial obstruction. Non-round, flattened inspiratory flow shape in nasal prongs signal is indicative to partial collapse in the upper airway (32–36). This flow limitation pattern is normally present during UARS events and hypopneas, when it is associated with frequent arousals (37). However, it also appears in prolonged periods up to several minutes (38). As flow limitation is associated with increasing negativity in pESO (39) and nasal pressure signal strongly correlates with pneumotachograph (40), it seems reliable to detect prolonged partial upper airway obstruction by nasal prongs. However, flow limitation may exist without upper airway obstruction (32) and vice versa (16). In addition, in some cases nasal prongs may even increase upper airway resistance (41). To conclude, flow limitation measured with nasal prongs is suggestive, but not a fully reliable marker of increased effort.

Particularly in the clinical PSG of children, prolonged partial obstruction is often detected by the surface electromyography (EMG) of the diaphragm and intercostal muscles, which seems to reflect changes in respiratory effort (42). However, frequent technical problems limit the use of EMG in assessing respiratory effort (43). There are no studies evaluating the usefulness of inductive belts in the diagnostics of prolonged partial obstruction. The method requires frequent calibration when position changes, which restrict its use (43, 44). Prolonged partial obstruction is usually associated with sustained crescendo snoring. Using a snoring signal to assess prolonged partial obstruction is, however, problematic, since there is no means to differentiate between benign snoring and snoring associated with marked partial obstruction.

Non-invasive techniques of measuring respiratory effort were reviewed recently by Vandenbussche et al. (45), but in Finland in conjunction with conventional sensors the most used sensors in detecting prolonged partial upper airway obstruction are mattress sensors: Static Charge Sensitive Bed (SCSB) and Electromechanical film transducer (Emfit) (46). In SDB diagnostics raw mattress signal reveals gross body movements. The low-frequency band (0.3–10 Hz) visualizes breathing movements, and the high-frequency band (6–16 Hz) shows heart activity and high-frequency spikes. Experimentally these spikes are shown to correlate with increasing respiratory effort (47).

The mattress breathing categories are visually scored by the three mattress channels. The categories with high-frequency spikes represent increased respiratory effort (10, 16). Normal breathing consists of regular breathing movements with no flow limitation, no high-frequency spikes and mostly normal pESO values. Obstructive periodic breathing is composed of periodic fluctuation of respiratory movements with periodically emerging high-frequency spikes and periodic pESO swings. Flow signal shows periodic respiratory events (apneas and hypopneas). IRR pattern represents prolonged partial upper airway obstruction. IRR consists of regular respiratory movements with sustained high-frequency spikes and sustained negative increase in the pESO signal (Fig. 2). Flow limitation pattern is commonly seen in the flow channel.

In clinical practice, PSG is not needed to detect partial upper airway obstruction. Periods longer than hypopnea (minimum 1–3 min) are used as indicative of sustained upper airway resistance. Prolonged partial obstruction is usually associated with sustained crescendo snoring. Combining these two key factors, measuring the flow limitation with nasal prongs and using snoring signal, together gives a reliable marker of prolonged partial obstruction (10, 15, 48). Key features of prolonged partial upper airway obstruction during sleep are presented in Table 2.

**Table 2:** Clinical key features of prolonged partial upper airway obstruction

Key features of prolonged partial upper airway obstruction	
Definition criteria	– Prolonged flow limitation for more than 20–30% of TIB or TST
	– Crescendo snoring
	– CO_2_ increase during sleep >6 kpa
	– Absence of repetitive arousals
Diagnostic findings	– Sustained flow limitation in nasal prongs
	– Increased respiratory effort (Emfit mattress signal, inductive plethysmography, esophageal pressure)
	– Crescendo snoring
Populations at risk	– Children
	– Females
	– Postmenopausal women
	– Patients with symptoms of SDB but with low AHI
	– Patients on antipsychotic therapy gaining weight
Symptoms	– Fatigue>sleepiness
	– Depressive symptoms
	– Morning headache
	– Decreased quality of life
	– Similar comorbidity as in SDB with high AHI
Treatment options	– CPAP
	– Mandibular advancement devices in supine position-dependent partial obstruction
	– Surgery unlikely to work

AHI=apnea–hypopnea index, CPAP=continuous positive airway pressure, CO_2_=carbon dioxide, SDB=sleep-disordered breathing, TIB=time in bed, TST=total sleep time.

## Clinical presentation

### Prevalence of prolonged partial upper airway obstruction

The prevalence of prolonged upper airway obstruction during sleep is not known. Short UARS events have been shown to represent only 5.3% of all obstructive non-apneic events in patients with moderate OSA (49). Based on limited number of small studies, prolonged upper airway obstruction seems to be more common than classical OSAS and have female predominance. Comparing 233 age- and body mass index (BMI)-matched male–female pairs with suspected OSAS, prolonged partial upper airway obstruction was the most common single breathing abnormality, accounting for half of all observed breathing abnormalities in women compared to only one third in men (12). From 157 consecutive patients referred to full PSG 29.9% presented with OSA, whereas 10.8% had prolonged partial obstruction with normal AHI (10). The proportion of females was higher in patients with prolonged partial obstruction as compared with OSA patients (47 and 17% females, respectively). In healthy postmenopausal women, the prevalence of partial upper airway obstruction was 10 times higher (17.7%) compared with that of frank OSA (1.6%) (13). In a clinical sample of pre- and postmenopausal women, the prevalence of SDB was 79.4 and 86.2%, respectively (31). The prevalence of frank obstructive sleep apnea did not differ, whereas partial upper airway obstruction was more prevalent in postmenopausal (66.1%) than in premenopausal (50.9%) women (31). A cross-sectional cohort study showed that in the ‘normal’ asymptomatic population the percentage of total sleep time with flow limitation is less than 30% in 95% of the population (50).

In the clinical sleep laboratory population, 10% had only partial upper airway obstruction and another 8% had OSA plus partial upper airway obstruction (10). In another clinical sleep laboratory population comparing age- and BMI-matched male–female pairs, women had 10.5% and men had 7.5% of partial upper airway obstruction (51).

### Symptoms and signs

In sleep studies, compared to men, women have lower AHI (52). Women with sleep apnea are frequently symptomatic with low AHI (53). This suggests that factors other than the AHI are likely to contribute to the symptoms of SDB among women. Lower AHI in women is contrasted with higher occurrence of prolonged partial upper airway obstruction (13, 31, 54, 55) which usually appears in SWS and is associated with elevated CO_2_ levels (14, 26). Increased CO_2_ during sleep might explain gender differences in symptom and comorbidity profiles (56, 57). This is supported by findings that excessive daytime sleepiness and daytime fatigue associate with habitual snoring independent of age, obesity, smoking, AHI, and sleep parameters in women (58). In a retrospective study of 240 women no difference was observed in terms of sleepiness or other symptoms between women with partial upper airway obstruction or classical obstructive sleep apnea (59). Sleep laboratory patients with prolonged partial obstruction had decreased life quality score as compared with OSA patients (10). Micrognathia was more common in women with partial upper airway obstruction compared to those with OSA (59).

### Impact of age and obesity

**Fig. 3:**
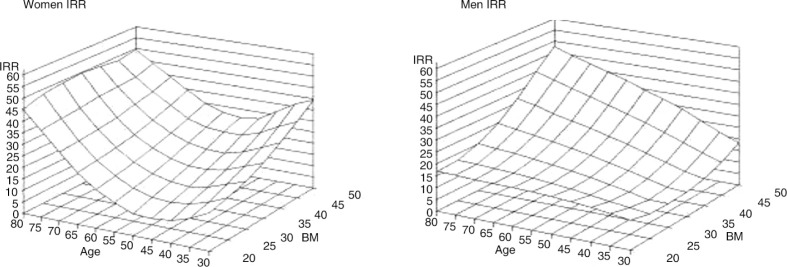
The three-dimensional figures demonstrate that the associations are non-linear and suggest that in women (left-hand panel) there is a consistently increasing susceptibility for prolonged partial upper airway obstruction after 65 years of age over the entire BMI range, whereas in men (right-hand panel) partial obstruction associates with the combination of high age–high BMI (51). (Reproduced with permission from Respir Physiol Neurobiol).

Prevalence of OSAS increases with age and obesity. In a study of 233 age- and BMI-matched male–female pairs, classical sleep apnea increased with increasing BMI only in men, whereas partial obstruction increased with moderate to morbid obesity in both genders after the age of 65 years (Fig. 3) (51). In women, increasing age was also associated with an increase in prolonged partial obstruction and showed tendency also in men (Fig. 3). These findings suggest that men have a gender-specific BMI-dependent predisposition for periodic obstruction, that is, classical OSA, which is likely to reflect gender differences in the ventilatory and upper airway control during sleep or in the arousal threshold. Moreover, AHI is likely to underestimate the impact of SDB especially in elderly patients.

Respiratory instability predisposes to periodic upper airway obstruction whereas stable breathing underlies partial obstruction. Hormonal changes at menopause may stabilize control of breathing, whereas increasing obesity could predispose to respiratory instability because of increasing sympathetic activity and carotid body gain (60).

## Comorbidity

Mild OSA in terms of AHI may not translate into less severe health consequences than OSA with higher AHI (11, 59, 61). Episodes of prolonged partial upper airway obstruction are associated with increased intrathoracic pressure variation (16) and increased CO_2_ level (14), indicating increased respiratory efforts and respiratory workload. Partial upper airway obstruction may be one important factor in the development of hypercapnic respiratory failure (62).

Case series suggest that hypertension might be a consequence of untreated partial obstruction (63, 64). In a study of 133 women and 122 men, reimbursed medication for hypertension was used three times more often in patients with partial obstruction compared with those with frank OSA (65). However, other co-morbidities with reimbursed medication did not differ between patients with partial obstruction compared with those with frank OSA except for asthma and/or COPD which was more prevalent among patients with partial obstruction (65). This indicates similar comorbidity in patients with prolonged partial upper airway obstruction and in those with OSA. On the other hand, the same study found a lower risk for hypertension in postmenopausal women presenting with predominantly partial upper airway obstruction compared with those with classical OSA (65). This is supported by diminished sympathetic tone measured with heart rate variability in IRR compared with OSAS patients (66).

In women with pre-eclampsia, SDB typically manifests as flow limitation with increased nocturnal CO_2_ but low AHI and an increased number of oxygen desaturations especially during rapid eye movement sleep. Blood pressure responses to episodes of obstructive apnea are augmented in normal pregnancy and further in preeclampsia. The increased sympathetic activity during the third trimester contributes to the increased prevalence of OSA, which in turn probably further augments sympathetic tone and predisposes to preeclampsia (67). Nasal CPAP treatment alleviated sleep-induced blood pressure increases and upper airway collapsibility (67) and improved fetal movement activity (68) in preeclampsia.

Depression is more frequently observed in female OSA patients compared with male patients which may at least partly explain the higher degree of daytime fatigue and sleepiness in female patients with low degree of OSA. Sforza et al. (69) reported that women with OSA had five-fold increased risk of having depression compared to men (11.9% vs. 2.7%). Depression did not correlate with AHI but it did with the time with SaO_2_ below 90% and with the mean SaO_2_. Episodes of partial obstruction are often associated with prolonged mild steady state lowering of the SaO_2_. Therefore it is possible that in the absence of correlation with AHI, partial obstruction (not measured in their study) could have contributed to these correlations. Fatigue and sleepiness are symptoms of depression but so are insomnia and anxiety. Depression is not a single entity but includes a variety of different phenotypes. Fatigue and sleepiness are common symptoms in atypical depression, but the biological mechanisms of these symptoms are not known. Partial obstruction with increased CO_2_ levels during sleep could be one such mechanism, since 12 weeks of therapy with CPAP controlling both AHI and partial obstruction even in women with moderate severity of sleep apnea results in improvement of quality of life, daytime sleepiness, mood state, anxiety, and depression symptoms (70).

## CPAP adherence

Partial upper airway obstruction should not be considered as ‘simple snoring’ or mild OSA, which are associated with lower adherence to nasal CPAP therapy (71). Partial upper airway obstruction, even in the absence of episodes of apnea or hypopnea, highly predicts good CPAP adherence and efficient relief of symptoms with nasal CPAP therapy (12, 72). Prolonged flow limitation has been evaluated in stepwise CPAP titration studies. The contour of inspiratory flow appears as the simplest variable that best correlates with lowest esophageal pressure during CPAP titration (73). Prolonged flow limitation also seems to be the earliest indicator of obstruction, even more sensitive than snoring, during decreases in CPAP pressure (74). Though treatment of symptomatic partial upper airway obstruction is not suggested by guidelines but it has been our clinical practice since the nasal CPAP became available (1987). This has been based on the knowledge about flow limitation (75) and recognition of prolonged partial obstruction with mattress sensors.

An AHI of more than five per hour is considered significant for diagnosis of OSA but often higher AHI is required for initiation of nasal CPAP therapy. Sleepy patients with low AHI, with non-apneic snoring should not be overlooked. Considering increased fatigue in women with OSA despite low or normal AHI, it has been suggested that treatment should be considered in symptomatic women with snoring or partial upper airway obstruction even if AHI<5/h (17, 58). This approach is supported by good CPAP adherence in patients with predominantly partial obstruction (12, 72). If the patient is compliant to the CPAP therapy and becomes symptomless during the follow-up, it is our clinical practice to continue with the CPAP therapy as a long-term treatment.

For women in particular, it is important that the CPAP is titrated to control not only obstructive sleep apnea but also episodes of partial upper airway obstruction. Recent female-specific autotitrating algorithms for CPAP devices may reduce flow limitation more efficiently but whether this results in better treatment outcome, remains to be seen (76).

## Conclusions

Particularly in women, the upper airway obstruction often manifests as non-countable non-apneic respiratory events (snoring, flow limitation or partial upper airway obstruction) (11, 12). Failure to recognize prolonged partial obstruction in sleep studies may lead to under recognition of SDB especially in women (56, 77). Partial obstruction, even in the absence of episodes of apnea, may cause clinically significant symptoms. Patients with symptomatic partial upper airway obstruction respond and adhere to nasal CPAP therapy at least as well as those with ‘conventional’ OSAS (12, 72). The scarce available data suggest that despite less severe OSA in females, the consequences of OSA might not be less severe. Therefore, identifying the phenotype of partial upper airway obstruction during sleep may lead to timely and appropriate therapy especially in female patients and might also reduce lost work days among them (78, 79).
